# Health care associated *Clostridium difficile* infections in University Hospital Trnava during last five years (2010 – 2014)

**DOI:** 10.1186/2047-2994-4-S1-P24

**Published:** 2015-06-16

**Authors:** A Streharova, M Garabasova, JS Brnova

**Affiliations:** 1 Department of Public Health, Trnava University, Trnava, Slovakia; 2 Department of Laboratory Medicine, School of Health Sciences and Social Work, Trnava University, Trnava, Slovakia; 3 Laboratory of Molecular Microbiology, St. Elisabeth University, Bratislava, Slovakia

## Introduction

In Slovakia, mandatory surveillance of Clostridium difficile infection (CDI) have been established due to electronically national Epidemiological information systems (EPIS) long time ago, but compliance to surveillance are low.

## Objectives

The aim of this hospital-based study was to assess real prevalence and outcome of CDI in University Hospital Trnava, Slovakia during years 2010-2014.

## Methods

We analysed all patients with laboratory confirmed CDI (RIDA®QUICK *Clostridium difficile* Toxin A/B immunochromatographic rapid assay, R-Biopharm) in University Hospital Trnava (618-bed; approx. 25 000 patients per year) from laboratory information system and medical records.

## Results

During five years period were identified 317 CDI with declining trend in recent years for 10 000 admission. Health care associated CDI (HA-CDI) accounted for 75,7% (240). The mean age of patients was 72,8 ± 15 years (range 15-96) and 60,3% (191) were women. The most frequently antibiotics used before onset CDI were quinolones (25,9%) for the treatment of respiration or urinary tract infection. Overall recurrence of CDI was observed within 3 months after the first episode in 6,9% (22) of cases. Total hospital mortality rate was 13,9% (44) and hospital mortality associated with CDI only 1,9% (6).

## Conclusion

These results show high prevalence HA-CDI in our hospital and emphasizing the importance of implementing better infection control practice in order to prevent further spread. To date no data about specific ribotype of *Clostridium difficile* and about prevalence in long-term care facility are available in Slovakia.

## Disclosure of interest

None declared.

